# Some Remarkable Cases That I Have Seen*Read at May Meeting Brazos Valley Medical Society, and voted to be published in The Texas Medical Journal.

**Published:** 1906-09

**Authors:** W. A. Bedford

**Affiliations:** Franklin, Texas, President Brazos Valley Medical Association


### TEXAS MEDICAL JOURNAL.

Established July, 1885.

F. E. DANIEL, M. D., - - - - Editor, Publisher and Proprietor.

PUBLISHED MONTHLY.—SUBSCRIPTION $1.00 A YEAR.

VOL. XXII. AUSTIN, SEPTEMBER, 1906. No. [illegible].3

The publisher is not responsible for the views of contributors.

For Texas Medical Journal.

### Some Remarkable Cases That I Have Seen.*

BY W. A. BEDFORD, M. D., FRANKLIN, TEXAS, PRESIDENT BRAZOS VALLEY MEDICAL ASSOCIATION.

I was called to see Joseph B., aged 11 years, after supper, in the month of February. He had a chill that afternoon about 3 o'clock. His temperature was about 104½°, his pulse 135, his breathing 34, his face was flushed, eyes suffused, and he complained of intense pain in his right side; coughed a good deal, but it was dry.

I examined and found the right lung very dull all over, with a few subcrepitant rales. My diagnosis was pneumonia. I went to see him next morning and found all the symptoms about the same, except perhaps slightly increased. He had the characteristic rusty sputum, well marked.

I called again the next morning and found his condition gradually growing worse; none of the symptoms having been ameliorated in the least. That night about 2 o'clock I was called to see him hurriedly, the messenger saying that he thought the boy was dying. When I arrived and examined him I did not think that he would live until morning. I aroused him by shaking him considerably, but he refused to take any medicine. I argued with him, and finally told him that I would give him a nickle if he would take it. He at once raised up and swallowed the dose. He soon afterward went off into an unconscious condition, and about an hour later the nickle was found in his mouth and removed. *Remember this,* I will refer to it again.

---

*Read at May Meeting Brazos Valley Medical Society, and voted to be published in The Texas Medical Journal.

At this time the boy was, to all appearances, dying. He could not be aroused, and would make no effort to swallow water or medicine if placed in his mouth. I now thought it would only be a matter of a few hours, at best, until the end.

The mother begged me to do something for her child. I said that there was no hope; but I thought that I would give him medicine hypodermically, and try to revive him. I gave him sixty-five drops of tincture of digitalis and one-fifteenth grain of strychnia, together with one drachm of whiskey, hypodermically. In about one hour I went home, but left word to call me if he was living at sunrise. They promptly sent for me at sunup, and said that he was no worse, but not any better. I found this to be so. He was still unconscious, but his pulse was slower and stronger. He could not swallow even water. I again gave digitalis (20 drops) and strychnia (1/30 grain), and repeated it every four hours that day. To make a long story short, I secured a man to stay with him and give him the digitalis and strychnia every four hours, day and night, hypodermically, and I saw him twice each day. Each time I saw him I did not think he could possibly live until the next day.

This boy lay in this condition just five days and nights, or 120 hours. He never swallowed a drop of anything; never moved without being moved by the nurse. His kidneys and bowels acted a few times, involuntarily. He never showed any signs of life at all except you could see him breathe. All of a sudden he opened his eyes and looked up at his nurse, just as if coming out of a sound sleep, and said, "Where is the nickle that Dr. Bedford gave me?" From this minute on he took water and food, and was perfectly rational, and made a rapid and perfect recovery.

As above stated, the only thing that he took to sustain life was the digitalis and strychnia, hypodermically, every four hours.

Case No. 2.—A woman called at my office one morning with her baby, about four months old, in her arms. She seemed very much alarmed, and said: "Doctor, what on earth can be the matter with my baby? It appears as if both of its legs and arms are broken, but it has had no fall or hurt in any way." On examination I found both hands and both feet hanging perfectly limp, falling from side to side, as though nothing but the skin was holding them together. There was no indication of pain whatever, and the little fellow was to all appearances comfortable. There was no swelling of the joints and no sign of inflammation. The history of the case was that it had come on during the night and was discovered that morning. My opinion was that it was a separation of the epipheses of the respective bones. Having never, however, seen a case of this character before, I called in a doctor friend to see it with me, and he acquiesced in my diagnosis.

I inquired into the history of the parents, and found that the father had been treated several years before that for syphilis. He was, to all appearance, healthy and gave no evidence of ever having been infected.

I placed this child on an anti-syphilitic course of treatment, and at the same time placed each of the limbs in a plaster dressing, and allowed them to remain six weeks. When I removed them union was perfect, and the child never had a bad symptom. It walked at the usual time, and, so far as physical appearances go, was a healthy and normal child.

Two years ago I was in Marlin one day and met the father, who said to me: "Doctor, I want you to see that boy of mine. He and his mother are right here in the store." I stepped in and met the mother, and the first thing that she said was: "Doctor, here is our boy. He has always been well since you treated him." The child was at that time 12 years old, and a fine specimen of physical strength. I will add that I kept the child on the medicine for nearly a year. This case was unique, so far as my practice goes. I have never seen a case like it since or before.

Case No. 3.—I was called one morning to see a negro man lying by the side of the railroad track just below town, who we supposed had been knocked from a freight train. He was profoundly comatose and perfectly insensible to all pain or noise. He was bleeding from the mouth and ears. A few cuts of minor character were on his face, a few bruises on his body, none serious. No broken limbs. A little below and in front of the left parietal eminence was a scalp wound in the form of a three-cornered cut, from which blood was oozing slightly. His pulse was regular and strong, beating about the normal rate; his temperature was normal; but his breathing was stertorous and labored. I could find no sign of injury to his lungs or bowels.

I had him carried to a vacant house near the home of another negro, who was very intelligent and withal a good-natured fellow. I had this man take care of the patient and watch him, for this was all that could be done by the nurse, as the man could not swallow anything at all, and never moved himself—not even a muscle. My idea was that his skull was fractured, and that he would soon die. I went to see him two or three times that day, and tried to give him water and milk, but he would make no effort to swallow. I told Dave, the nurse, to stay with him through the night, which he did; but when I called next morning, the man showed no change whatever for better or worse.

He remained about the same throughout the second day and night. I could see no difference at all in his condition. That evening I thought he would not live until morning. I called again the third morning, and found patient about the same as the day before. He had never shown any symptoms of rallying—I could not see that he was any better or any worse. About 3 p. m. on the third day I went to see him, and finding him still living, and in about as good condition as he had been, I determined to trephine his skull.

I asked Dr. Blalock to go with me and see him. He concurred in the opinion with me that there was everything to gain and nothing to lose by performing this operation. Accordingly, we trephined at the point of the scalp wound, removing a large button of bone. There was no clot found on the brain, and when the piece of bone removed was examined carefully there was no sign whatever of a fracture, and we considered our operation a labor in vain. We dressed the wound, however, and had the nurse stay with him again that night, supposing, of course, that he would pass away during the night, because we expected nothing from what we had done.

Imagine my surprise when I called the next morning and found my patient sitting up and eating a regular meal, furnished by the nurse, and, above all, conversing rationally and intelligently with the nurse, who had been watching him all this time. The nurse told me that about sunup that morning the man seemed to wake up as if out of a natural sleep, and began talking to him. He asked for water and food, the latter of which he was eating when I arrived. He seemed to enjoy his breakfast hugely, and when he was through he told me all about how the accident occurred; where he was from, his name, etc. He said that he was stealing a ride and the brakeman, he supposed, hit him on the head with something, and that was the last that he remembered. This man improved very rapidly, and in a few days the town made up money and sent him to his people, who lived at Gause, in Milam county.

This case, to my mind, was a remarkable one. I have never been able to account for the trephining doing him any good, but I suppose that it did, at least, he never did get any better until after the operation.

Case No. 4.—While eating breakfast one morning, I heard some one hollo, "hello!" at my gate. I got up from the table and walked to the door. A Mexican I knew well was standing at the gate. He said to me, "Good morning, doctor, I would like to see you a minute." I stepped out of the house and walked to where he was. I said, "Amelia, what do you want?" He said, "Doctor, I am shot, and I want to see if you can do anything for me." I said, "Where are you shot?" and he said, "I am shot in the mouth, doctor." I said, "When were you shot?" He said, "Three nights ago; had not you heard about it?" I said, "No; open your mouth and let me see." He opened his mouth and I could plainly see where the ball had entered the tongue a little to the right side of the median line of the tongue. I said, "Where did it go to?" He at once turned the back of his neck to me, and said, "Here is where it came out," pointing to a place just to the left of the spinal column on a level with the base of the tongue. I looked at him a moment in amazement, and then said, "You don't need any doctor; if that shot wouldn't kill you in three days you need not be afraid of dying."

The man then gave me a history of his case, as follows: He said that he was at a negro dance and was drinking, and decided that he would have some fun. He took out his six-shooter, which was a forty-four-caliber Colt's, and began to snap it around promiscuously, thinking that it was not loaded at all. Some of the negroes grabbed him and tried to get him to put the pistol up, saying, "You will shoot somebody directly." He told them, "No, there was no danger in that gun; that he could eat one of that size." And at this time he turned the pistol toward himself, opened his mouth and put the muzzle in it and pulled the trigger and it went off, the ball passing through the whole length of his tongue and out at the back of his neck, as detailed above.

The shot did not knock him down, he said, but he was very much frightened, and threw the pistol down, and ran about three hundred yards to a negro's house and went in and told them about it. He sat around for an hour or two and then went to bed. He got up next morning and did not remain in bed a minute on account of the injury. I afterward talked with the negro at whose house it occurred; he corroborated everything that the Mexican had told me.

In concluding this case, I wish to say that if I had not seen it, I do not know whether I could have believed such a thing could occur without killing the man, and I shall not think hard of any one who refuses to believe my story. To me it seems very close to the borders of the impossible.

